# Supplementary material for: Identification of photoperiod-induced specific miRNAs in the adrenal glands of Sunite sheep (Ovis aries)
Source: Front Vet Sci. 2022 Jul 22;9:888207. doi: 10.3389/fvets.2022.888207 (PMC9354845; doi:10.3389/fvets.2022.888207)
Supplement: Supplementary file 1 [file Data_Sheet_1.ZIP › Supplementary file/miRDeep2 file.zip!/miRDeep2 file/Know_Structure/SP42A2/oar-let-7g.pdf]

miRBase precursor : oar-let-7g
Total read count : 798234
oar-let-7g read count : 798219
remaining reads : 15

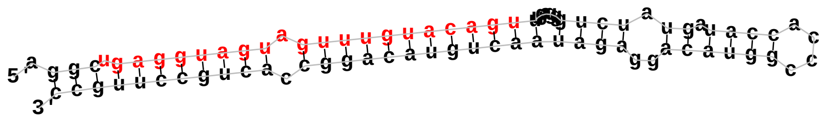

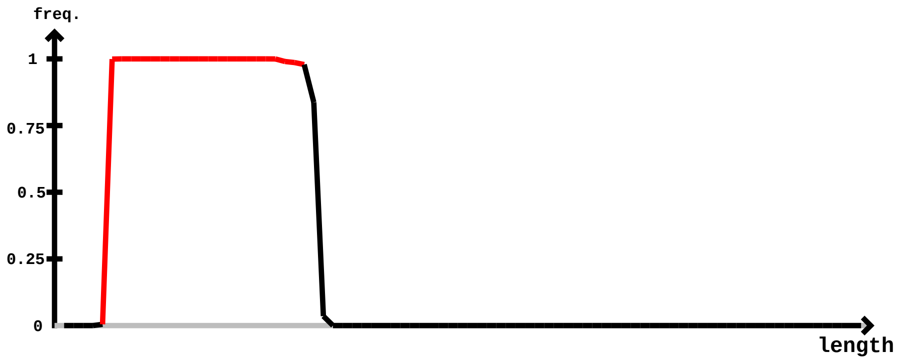

oar-let-7g

```
5'- aggcugagguaguaguuuguacaguuugagggucuaugauaccacccgguacaggagauaacuguacaggccacugccuugcc  -3'    exp
    .(((.((((((((.((((((((((((.....((((.((.((((....))))))..)))))))))))))))).)))))))))))     reads      mm         sample
    ..gcugagguaguaguuuguacaguuu........................................................     1          0          seq
    ...cugagguaguagAuugu...............................................................     2          1          seq
    ...cugagguaguaguuugu...............................................................     2          0          seq
    ...cugagguaguaguuugua..............................................................     2          0          seq
    ...cugagguaguaguuuguac.............................................................     49         0          seq
    ...cugagguaguaguuuguaca............................................................     25         0          seq
    ...cugagguaguaguuuguacag...........................................................     30         0          seq
    ...cugagguaguaguuuguacagu..........................................................     796        0          seq
    ...cugagguaguaguuuguacaguu.........................................................     2715       0          seq
    ...cugagguaguaguuuguacaguuu........................................................     110        0          seq
    ....ugagguaguaguuuAua..............................................................     1          1          seq
    ....ugagguaguaguuugua..............................................................     225        0          seq
    ....ugUgguaguaguuugua..............................................................     1          1          seq
    ....ugagguaguaguuuguac.............................................................     7497       0          seq
    ....ugagguagCaguuuguac.............................................................     15         1          seq
    ....ugagguaguaguuuguaca............................................................     3187       0          seq
    ....ugagguaguaguuuguacag...........................................................     5865       0          seq
    ....ugagguaguaguuuguacagu..........................................................     112044     0          seq
    ....ugagguaguaguuuguacaguu.........................................................     636961     0          seq
    ....ugagguaguaguuuguacaguuu........................................................     28149      0          seq
    ....ugagguaguaguuuguacaguuug.......................................................     14         0          seq
    ....ugagguaguaguuuguacaguuuga......................................................     1          0          seq
    .....gagguaguaguuuguac.............................................................     2          0          seq
    .....gagguaguaguuuguaca............................................................     3          0          seq
    .....gagguaguaguuuguacag...........................................................     3          0          seq
    .....gagguaguaguuuguacagu..........................................................     78         0          seq
    .....gagguaguaguuuguacaguu.........................................................     416        0          seq
    .....gagguaguaguuuguacaguuu........................................................     21         0          seq
    ......agguaguaguuuguacaguu.........................................................     4          0          seq
    ...........................................................aacuguacaggccacugccuugc.     1          0          seq
    ............................................................acuguacaggccacugccuugc.     1          0          seq
    .............................................................cuguacaggccacugccuugc.     8          0          seq
    .............................................................cuguacaggccacugccuugcc     5          0          seq
```
